# *De novo* cholesterol biosynthesis: an additional therapeutic target for the treatment of postmenopausal breast cancer with excessive adipose tissue

**DOI:** 10.37349/etat.2022.00116

**Published:** 2022-12-28

**Authors:** Danila Coradini

**Affiliations:** Department of Clinical Sciences and Community Health, Campus Cascina Rosa, University of Milan, 20133 Milan, Italy; Istituto Nazionale Tumori “Fondazione Pascale” Via Mariano Semmola, Italy

**Keywords:** Breast cancer, cholesterol biosynthesis, statins, metformin

## Abstract

The onset and development of breast cancer in postmenopausal women are associated with closely related individual-dependent factors, including weight gain and high levels of circulating androgens. Adipose tissue is the most peripheral site of aromatase enzyme synthesis; therefore, the excessive accumulation of visceral fat results in increased androgens aromatization and estradiol production that provides the microenvironment favorable to tumorigenesis in mammary epithelial cells expressing estrogen receptors (ERs). Moreover, to meet the increased requirement of cholesterol for cell membrane assembly and the production of steroid hormones to sustain their proliferation, ER-positive cells activate *de novo* cholesterol biosynthesis and subsequent steroidogenesis. Several approaches have been followed to neutralize the *de novo* cholesterol synthesis, including specific enzyme inhibitors, statins, and, more recently, metformin. Cumulating evidence indicated that inhibiting cholesterol biosynthesis by statins and metformin may be a promising therapeutic strategy to block breast cancer progression. Unlike antiestrogens and aromatase inhibitors (AIs) which compete for binding to ER and inhibit androgens aromatization, respectively, statins block the production of mevalonic acid by inhibiting the activity of 3-hydroxy-3-methylglutaryl-coenzyme A (HMG-CoA) reductase, and metformin hampers the activation of the sterol regulatory element-binding protein 2 (SREBP2) transcription factor, thus inhibiting the synthesis of several enzymes involved in cholesterol biosynthesis. Noteworthy, statins and metformin not only improve the prognosis of overweight patients with ER-positive cancer but also improve the prognosis of patients with triple-negative breast cancer, the aggressive tumor subtype that lacks, at present, specific therapy.

Epidemiological evidence indicates that the onset and development of breast cancer in postmenopausal women are associated with some closely related individual-dependent factors: weight gain, metabolic syndrome, and high levels of circulating androgens. After menopause, the testosterone produced by the adrenal gland induces and supports the accumulation of adipose tissue in the abdomen [1], favoring the onset of insulin resistance. In its turn, insulin resistance inhibits the liver production of sex hormone-binding globulin (SHBG), which works by binding and sequestering free testosterone, and leads to an increase in circulating testosterone and a further accumulation of abdominal fat [2].

Because adipose tissue is the most peripheral site of aromatase enzyme synthesis, the excessive accumulation of visceral fat results in increased androgens aromatization and estradiol production, which provides a favorable stimulus to tumorigenesis in mammary epithelial cells expressing the estrogen receptor (ER) [3]. In addition, ER-positive cells activate *de novo* cholesterol biosynthesis and subsequent steroidogenesis to meet the increased requirement of cholesterol for cell membrane assembly and the production of steroid hormones to sustain their proliferation [4]. Indeed, cholesterol participates in several crucial cellular processes: it is an essential component of cell membranes, contributing to their packing and permeability; it serves as a precursor for several biochemical pathways, including the synthesis of steroid hormones, bile acids, and vitamin D, and it is involved in cell signaling processes. Indirectly, it assists the formation of lipid rafts, the plasma membrane structures where receptor proteins are near a high concentration of their specific ligands; directly, it activates the estrogen-related receptor α (ERRα), a transcription factor that acts as an energy sensor in controlling cellular adaptation under metabolic stress conditions [5].

## Antiestrogens and aromatase inhibitors: the conventional treatment for ER-positive breast cancer

Usually, two different therapeutic approaches are applied to reduce the stimulatory effect of estrogens on ER-positive breast cancer: (1) the competition for the binding to the ER using antiestrogenic compounds; (2) the inhibition of the aromatization of androgens into estrogens using specific inhibitors.

Since the early 1970s, both approaches have been adopted, even though the treatment with antiestrogens, specifically tamoxifen, was considered the treatment of choice until the late 1990s, when it was replaced, as the first-line therapy, by aromatase inhibitors (AIs) to overcome the tumor resistance occurring during the long-term adjuvant therapy [6], and in the treatment of metastatic ER-positive breast cancer.

The currently in use third-generation of AIs comprise two main classes of compounds. Type I AIs, such as formestane and exemestane, are androgen analogs that bind competitively but irreversibly to the enzyme. Type II AIs, such as anastrozole and letrozole, are reversible aromatase inactivators that fit into the substrate binding site of the enzyme.

Despite the recognized superiority of AIs over tamoxifen, in both early and metastatic ER-positive breast cancer (Table 1), clinical trials pointed out the occurrence of cancer resistance during the long-term adjuvant therapy with AIs that led to disease progression, especially in overweight patients. Thus, in the Arimidex, Tamoxifen, Alone or in Combination (ATAC) trial [7], although women receiving anastrozole had a lower recurrence rate than those receiving tamoxifen in all body mass index (BMI) classes, the benefit of anastrozole decreased significantly in women with a BMI > 30 kg/m^2^, when compared with women with a BMI < 23 kg/m^2^, both for overall recurrences [hazard ratio (HR) = 0.84; 95% confidence interval (CI) = 0.61–1.14 *vs.* HR = 0.64; 95% CI = 0.45–0.91] and distant metastases (HR = 0.96; 95% CI = 0.68–1.36 *vs.* HR = 0.59; 95% CI = 0.39–0.89). Austrain Breast & Colorecta Cancer Study Group-12 (ABCSG-12) trial [8], where premenopausal patients received adjuvant anastrozole, showed a similar finding. Overweight patients had a 60% increased risk of recurrence (15.1% *vs.* 9%, respectively; *P* = 0.02), and more than a doubling in the risk of death (HR = 2.14; 95% CI = 1.17–3.92) compared with normal weight patients. The retrospective analysis of the German Breast Cancer Care under Evidence-Based Guidelines (BRENDA) cohort that investigated the correlation among BMI, recurrence-free survival (RFS), and adjuvant endocrine therapy [9], confirmed the findings from clinical trials. It demonstrated that the efficacy of the treatment with AIs depended on BMI, suggesting that the total volume of the fatty mass results in an increased production of aromatase and, consequently, in the augmented conversion of androgens to estrogens, which, in turn, fuel ER-positive cells proliferation.

**Table 1.** Randomized trials of tamoxifen *vs*. AIs in the adjuvant breast cancer setting

**Clinical trial comparison(s)**	***N.* of cases(menopausal status)**	**Follow (years)up**	**Overall results**	**Predictive effects by BMI**	**References**
ATAC A *vs*. T	4,939 (post)	8.3	A better than T	A better than T at all BMI levels A less effective when BMI ≥ 30 for DDFS A relative benefit of A *vs.* T when BMI low	[7]
TEAM E *vs*. T → E	4,700 (post)	5.1	E = T 95% CI (0.39–0.84)	E better than T (2.75 years) at all BMI levels, significant in BMI > 30 (DDFS HR = 0.57, 95% CI = 0.39–0.84) E better than T → E (5.1 years) for any BMI (ns), BMI > 30 (DDFS HR = 0.75, 95% CI = 0.56–1.01 and OS HR = 0.71, 95% CI = 0.51–1.01)	[10]
ABCSG-12 A + G *vs*. T + G	1,684 (pre)	5.2	A + G = T + G (DFS) A + G worse than T + G (OS)	BMI < 25: A = T (DFS, OS) BMI ≥ 25: A worse than T HR = 1.49, 95% CI = 0.93–2.03 (DFS) HR = 3.03, 95% CI = 1.35–6.82 (OS)	[8]
BIG 1–98 L *vs*. T *vs*. L → T *vs*. T → L	4,760 (post)	8.7	L better than T (DFS, DDFS, OS) L → T or T → L = L	Treatment by BMI interactions (ns) L *vs*. T HR: 0.77 NW, 0.68 OW, 0.78 OB	[11]

*N.*: number of cases; A: anastrozole; T: tamoxifen; L: letrozole; E: exemestane; G: goserelin; T → E: tamoxifen followed by exemestane; L → T: letrozole followed by tamoxifen; T → L: tamoxifen followed by letrozole; BIG 1–98: breast international group 1–98 trial; NW: normal weight; OW: overweight; OB: obese; DDFS: distant disease-free survival; DFS: disease-free survival; OS: overall survival; ns: not significant

In light of this clinical evidence, it is clear that to improve the prognosis of overweight patients with ER-positive cancer, the conventional treatment with AIs, alone or in combination with antiestrogens, must be integrated with therapeutic strategies that reduce the production of estrogen through mechanisms alternative to androgens aromatization.

## Alternative therapeutic approach: inhibition of the enzymes involved in estrogens biosynthesis

An approach alternative to androgens aromatization implies the inhibition of 17β-hydroxysteroid dehydrogenase type 1 (17β-HSD1) and steroid sulfatase (STS), two enzymes involved in the synthesis of estrogens. 17β-HSD1 catalyzes, with high efficiency, the conversion of weakly active estrone into potent estradiol, whereas STS converts steroid sulfates like estrone sulfate and dehydroepiandrosterone sulfate to estrone and dehydroepiandrosterone (DHEA), respectively. Estrone and DHEA enter the synthesis of more potent estrogens and androgens that may fuel hormone-sensitive breast cancer cells.

Several classes of 17β-HSD1 inhibitors have been investigated in the past years, most of them having a steroidal structure [12] and, more recently, some potent nonsteroidal 17β-HSD1 inhibitors have also been discovered by virtual high-throughput screening [13]. Unfortunately, despite the promising results from preclinical studies [14–16], none reached clinical trials.

Conversely, among the several potent STS inhibitors designed and developed over the past 30 years [17], irosustat (STX64, BN83495), a tricyclic sulfamate ester, is the only one to have completed a phase I clinical trial against ER-positive metastatic breast cancer in postmenopausal women [18]. Irosustat was able to block almost completely STS activity in peripheral blood lymphocytes and tumor tissues. Besides, the inhibition of STS activity by irosustat was associated with significant reductions in serum concentrations of androstenediol and estrogens. However, despite the success in the phase I clinical trial, irosustat failed the phase II study, likely due to the impact on cancer cell growth. Proliferation studies showed that, in breast cancer cells treated with this STS inhibitor, the DNA synthesis decreased by a modest 20%, and the estradiol concentration decreased by 26%, suggesting the treatment with STS inhibitors alone was not sufficient to block cancer cells growth [19].

Since experimental evidence showed that the simultaneous inhibition of STS and 17β-HSD1 enzymes was more effective than blockage of each protein alone [19], a new family of steroid derivatives with dual inhibiting actions was designed and synthesized. Some of them are under biological evaluation [20, 21].

Another interesting class of compounds with dual inhibiting actions is the series of dual aromatase-sulfatase inhibitors (DASIs) in which, by various linker systems, the core motif of an STS inhibitor conjugated with the inhibitory pharmacophore of an AI, leading to hybrid structures with enhanced inhibitory activity.

To date, five structural classes of nonsteroidal DASIs have been developed: (1) derivatives of the nonsteroidal AI 4-{(4-bromobenzyl)-[1,2,4]triazol-4-ylamino}benzonitrile; (2) derivatives of letrozole; (3) derivatives of anastrozole; (4) derivatives based on a biphenyl template; and (5) a series of compounds with a hybrid structure of experimental DASIs [22]. When evaluated using an *in vitro* model (JEG-3 cells), several DASIs showed better aromatase or STS inhibitory activity than the single agent. The most potent DASI until now discovered is an imidazole derivative (DASI 25) with half maximal inhibitory concentration (IC_50_) values against aromatase and STS (0.2 nmol/L and 2.5 nmol/L, respectively) similar to those of letrozole (IC_50_ = 0.89 nmol/L) and irosustat (IC_50_ = 1.5 nmol/L), obtained from the same cell assay [22]. Nevertheless, it is yet to be demonstrated whether this high *in vitro* potency of DASI 25 can be reproduced *in vivo*, where the efficacy of a drug is affected by various pharmacokinetic parameters.

Among the DASIs until now studied *in vivo*, DASI 43 was the most potent. In adult female Wistar rats, it reduced pregnant mare serum gonadotropin (PMSG)-induced plasma estradiol levels by 92% and liver STS activity by 98% already three hours after dosing [23]. However, no DASI is under clinical evaluation at present.

## Cholesterol biosynthesis

**Figure 1.**
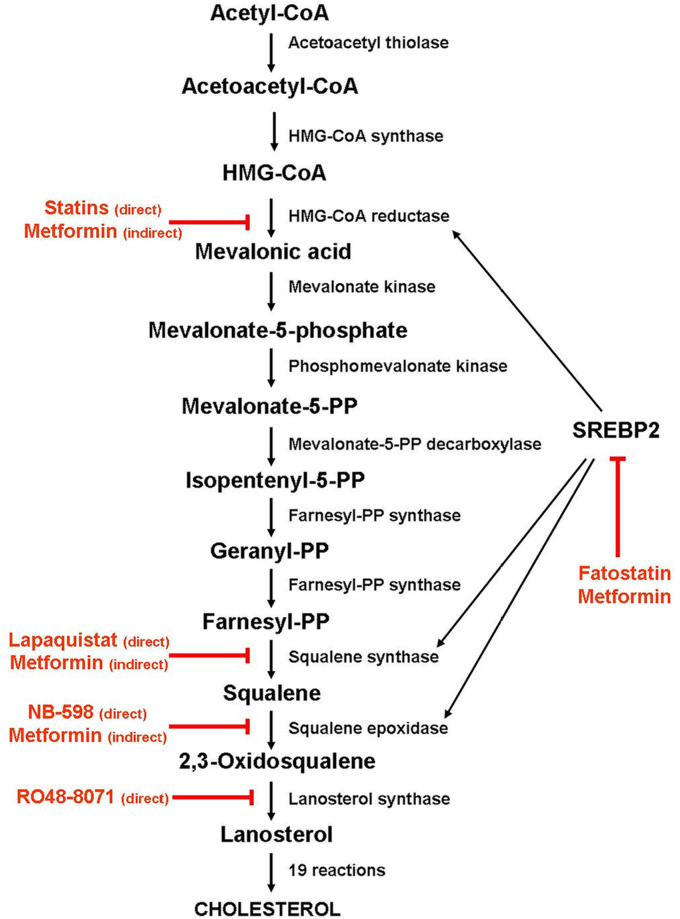
Schematic diagram depicting the enzymes involved in the cholesterol biosynthesis and the steps inhibited by statins and metformin. Black arrow: activation; red arrow: inhibition

As shown in Figure 1, cholesterol biosynthesis is a complex process that begins with the condensation of two molecules of acetyl-coenzyme A (acetyl-CoA) by the acetyl-CoA acetyltransferase to form acetoacetyl-coenzyme A (acetoacetyl-CoA), followed by the condensation of a third molecule of acetyl-CoA to form 3-hydroxy-3- methylglutaryl-coenzyme A (HMG-CoA). Then, HMG-CoA is reduced to mevalonic acid by HMG-CoA reductase (HMGCR). Due to a sterol-sensing domain, through which cholesterol functions as a negative regulator, HMGCR controls the production of mevalonic acid, acting as the first recognized rate-limiting enzyme for cholesterol synthesis. For this reason, HMGCR is the main target of statins, a class of cholesterol-lowering drugs, which work by blocking the access of HMG-CoA to the catalytic site of the enzyme [24]. Through a series of intermediate steps that form the so-called mevalonate pathway, mevalonic acid converts into isopentenyl-5-pyrophosphate (IPP), which, in turn, is transformed into geranyl-pyrophosphate (PP) and then into farnesyl-PP by farnesyl-PP synthase.

By condensing two farnesyl-PP molecules, squalene synthase forms squalene, which is then converted into squalene-2,3-epoxide by squalene epoxidase (SQLE). Because SQLE is the enzyme that commits cholesterol biosynthesis irreversibly, this reaction has been recognized as the second limiting step of the biosynthetic process. Once synthesized, squalene-2,3-epoxide cyclizes to form lanosterol, which converts into cholesterol through intermediate reactions.

Alternatively, farnesyl-PP can be transformed, by geranylgeranyl-PP synthase, into geranylgeranyl-PP, a non-sterol isoprenoid derivative that, like farnesyl-PP, can modify post-transcriptionally (geranylation and farnesylation, respectively) and target small GTPases, such as Ras proteins, to their site of action [25, 26].

Cholesterol biosynthesis is regulated mainly by the sterol regulatory element-binding protein 2 (SREBP2), a transcription factor that controls the expression of most genes involved in the biosynthetic pathway, including *HMGCR*, squalene synthase [farnesyl-diphosphate farnesyl transferase 1 (*FDFT1*)] and *SQLE*. In the presence of cholesterol, the SREBP2 inactive precursor resides in the endoplasmic reticulum membrane, where it binds to the SREBP cleavage-activating protein (SCAP), a cholesterol-sensing protein that forms a ternary complex with insulin-induced gene 1 (INSIG1), an endoplasmic reticulum-anchored protein. Under cholesterol-deprivation conditions, INSIG1 and SCAP binding disrupts, SCAP undergoes a conformational change, and the SCAP/SREBP2 complex moves to the Golgi apparatus, where the SREBP2 precursor is cleaved in a two-step process. The N-terminal half of the protein, which corresponds to the mature transcription factor, is then released in the cytoplasm and migrates to the nucleus, where it binds to the sterol regulatory elements (SREs) in the promoters of its target genes, including *HMGCR* and *SQLE* [27].

Experimental evidence indicated that SREBP2 is markedly upregulated in various cancers, including breast cancer, leading to the intracellular accumulation of cholesterol [28].

### Cholesterol biosynthesis as a target of anticancer therapy

*De novo* cholesterol synthesis can be inhibited by blocking the activity of the enzymes that play an essential role in the biosynthesis pathway. The first identified and the best studied of them is HMGCR, though squalene synthase, SQLE (also known as squalene monooxygenase), and lanosterol synthase (also known as 2,3-oxidosqualene cyclase), all distal to HMGCR, have been identified as potential targets for anticancer therapy [29–32]. While HMGCR is the main target of statins, squalene synthase, SQLE, and lanosterol synthase proved to be the targets of several nonstatin inhibitors.

Several classes of potential squalene synthase inhibitors have been investigated, but so far, only lapaquistat acetate (TAK-475), a benzoxazepine derivative, has been evaluated in advanced clinical trials, alone or in combination with statins. Phase II and III trials indicated that lapaquistat lowered low-density lipoprotein (LDL) cholesterol in a dose-dependent manner. However, adverse events such as the elevation of several liver enzymes levels, probably because of the conversion of farnesyl-PP into toxic farnesol-derived dicarboxylic acid, and upregulation of LDL receptors, halted lapaquistat’s clinical development [29].

As regards SQLE, there is some evidence that it could be a promising alternative to old targets in hypercholesterolemia therapy since the enzyme’s inhibition does not cause a cytotoxic effect [30]. Several potent SQLE inhibitors, including allylamines (FR194738, NB-598), squalene analogs (trisnorsqualene alcohol), and some natural compounds of selenium and tellurium, have been found. These SQLE inhibitors decrease LDL levels in a dose-dependent manner in HepG2 cells, L6 myoblasts, rats, hamsters, and dogs similarly or even higher than statins [31]. However, no SQLE inhibitor has been entered into clinical trials, due to the toxic effects, such as dermatitis and neuropathy observed in animal models.

Several lanosterol synthase inhibitors have been identified, among which RO48-8071 is the most potent [32]. Significantly, *in vitro* studies indicated that RO48-8071 greatly reduced ER-positive human breast cancer cell viability and prevented tumor growth when administered to mice with BT-474 tumor xenografts, with no apparent toxicity. Noteworthy, RO48-8071 did not affect the viability of normal human mammary cells. Since it degraded ERα while concomitantly inducing the anti-proliferative ERβ, it has been suggested that the anti-tumor properties of RO48-8071 are in part due to an off-target effect that increases the ratio of ERβ/ERα in breast cancer cells [33]. Despite these promising results, no clinical study has yet been activated.

### Statins and inhibition of the HMGCR activity

*In vitro* studies indicated that statins can inhibit cancer cell proliferation, invasion, and colony formation and induce apoptosis to suppress tumorigenesis, tumor survival, angiogenesis, and metastasis by regulating multiple signaling pathways [34]. In particular, statins act by inhibiting the catalytic activity of HMGCR, leading to the substantial reduction of mevalonic acid and depletion of the downstream intermediate products of the pathway, including farnesyl-PP and geranylgeranyl-PP, the two non-sterol isoprenoid derivatives essential for proper prenylation of the small GTPases such as Ras proteins [35], and squalene, which plays a key role in the irreversible commitment to cholesterol biosynthesis.

A growing body of clinical evidence indicated that statins reduce the incidence of breast cancer, the risk of disease recurrence (including contralateral cancer), and disease-specific mortality. In a meta-analysis of 10 studies [36], statins use was associated with improved RFS (HR = 0.64; 95% CI = 0.53–0.79) and significantly improved OS (HR = 0.66; 95% CI = 0.44–0.99) also in terms of breast cancer-specific survival (BCSS; HR = 0.84; 95% CI = 0.70–0.99). Similarly, a study performed on 52,723 women with non-metastatic breast cancer from the Danish Breast Cancer Group database [37] showed a reduced risk for contralateral breast cancer (CBC; HR = 0.64; 95% CI = 0.43–0.96), especially in the subgroup of patients assuming statins for more than 5 years. A positive association between statin use and a better prognosis was observed even in postmenopausal patients treated with AIs.

Of particular relevance is the finding that the positive association between statin use and the prognosis was also observed in triple-negative breast cancer (TNBC), the aggressive tumor subtype with a high propensity for metastatic progression and lacking specific treatment. Thus, a significant improvement of BCSS (HR = 0.42; 95% CI = 0.20–0.88) and OS (HR = 0.70; 95% CI = 0.50–0.99) was observed in patients with TNBC who started statin therapy in the 12 months after breast cancer diagnosis [38]. As for patients with no-TNBC, statin use was associated with a reduced risk for CBC (HR = 0.67; 95% CI = 0.45–1.00) [39].

However, clinical experience indicated that not all statins provide the same benefit for RFS which is restricted to lipophilic statins such as fluvastatin, atorvastatin, and lovastatin [40], because of their higher ability to penetrate cell membranes and reach intracellular targets.

Besides, despite good tolerance and therapeutic efficacy, some breast tumors proved to be resistant to lipophilic statins. Preclinical studies aimed to uncover the cause of such a resistance showed that the sensitivity of breast cancer cell lines to statin treatment inversely correlated with HMGCR mRNA and protein expression levels [41, 42] and that cell sensitivity can be acquired by the knockdown of the *HMGCR* gene before statin treatment [43]. Furthermore, a translational trial [44] investigating the effect of short-term, high-dose atorvastatin treatment on intratumoral cholesterol homeostasis of breast cancer patients showed that, after treatment, the expression of LDL receptor (LDLR) significantly increased in response to the negative feedback activated by the cholesterol depletion caused by the block of the mevalonate pathway and aimed to supply the required level of cholesterol through increased uptake of the circulating cholesterol-rich LDL.

### Fatostatin and SREBP2 inactivation

Fatostatin, a diarylthiazole derivative formerly termed 125B11, was first reported to inhibit the insulin-induced adipogenesis of 3T3-L1 cells and the serum-independent growth of human androgen-independent prostate cancer (DU145) cells [45]. Subsequent studies [46] have shown that in tissue culture, fatostatin impairs the activation of SREBP transcription factors, inhibiting their ER-to-Golgi translocation by binding to the escort protein SCAP at a different site from the sterol-binding domain. Consequently, SREBPs do not move into the nucleus and do not activate the expression of SREBP-responsive genes, including those involved in cholesterol biosynthesis. Furthermore, *in vivo* experiments showed that fatostatin lowers hyperglycemia and blocks fat accumulation in obese ob/ob mice [46].

Because of these promising results, fatostatin was explored as a therapeutic agent against cancer in different solid tumors, including breast cancer [47], where it inhibited growth and induced apoptosis in ER-positive cells and xenograft tumors in the mammary glands of mice. Although pre-clinical, these encouraging findings indicate that inhibiting SREBP activation by fatostatin could represent an alternative approach against ER-positive breast cancer, especially in patients with excessive adiposity.

### Metformin and inhibition of cholesterol biosynthesis

An increasing number of epidemiological studies have indicated that, besides the antidiabetic effect, metformin reduces both the incidence and mortality of several types of cancer, including breast cancer where metformin use was associated with a lower incidence of invasive breast cancer in postmenopausal women with type 2 diabetes [48] and premenopausal women with components of metabolic syndrome [49]. A meta-analysis indicated that in postmenopausal women with diabetes associated metformin use with a 30% lowered overall incidence of cancer when compared with patients with diabetes receiving other therapies [50]. Subsequent clinical studies demonstrated metformin effectiveness also in obese non-diabetic patients with breast cancer. In the phase II randomized trial aimed to evaluate the benefit of metformin treatment, the metformin-treated group showed a significantly higher decline in glucose level that increased with the increase in BMI [51].

Among the mechanisms proposed to explain the antitumor effect of metformin there are: (1) the suppression of hepatic gluconeogenesis, which results in the downregulation of the insulin/insulin-like growth factor I (IGF-I) pathway [52]; (2) the activation of the AMP-activated protein kinase (AMPK) pathway with resultant inhibition of crucial signaling pathways, such as those involving mammalian target of rapamycin (mTOR), human epidermal growth factor receptor-2 (HER2), and nuclear factor kappaB (NF-κB) [53, 54]; (3) the reduction of cellular cholesterol content [55], primarily by the AMPK-mediated block of SREBP2 activity by suppressing its cleavage process and nuclear translocation, and the subsequent inhibition of the downstream target genes expression [56].

However, while the genes involved in cholesterol biosynthesis were downregulated because of the metformin-induced block of the SREBP2 transcriptional activity, the expression level of *LDLR* gene increases with an increased level of the corresponding protein [57].

It is interesting to note that, in addition to the direct effects on tumor cells, metformin has proved to decrease the stromal production of estrogens by reducing the number of the M2-like macrophages, a subpopulation of protumor macrophages present in high quantities in inflamed breast adipose tissue forming the so-called crown-like structures (CLS). The presence of these structures was associated with the activation of NF-κB and the production of proinflammatory mediators such as necrosis factor alpha (TNF-α) and interleukin-1beta (IL-1β), paralleled by elevated levels of aromatase expression [58]. Because the severity of breast inflammation, defined as the CLS index, is correlated with BMI, the finding that metformin can reduce the number of aromatase-overexpressing macrophages is an attractive suggestion for the treatment of overweight patients with elevated levels of circulating testosterone [59].

## Conclusions

Because of the crucial role played in both membrane assembly and steroid hormone production, cholesterol biosynthesis appears as a promising therapeutic strategy alongside the conventional treatment with antiestrogens and/or AIs, especially in overweight postmenopausal women with high levels of circulating testosterone and in TNBC for which, at present, there is no specific therapy. In particular, HMGCR and the set of enzymes downstream in the mevalonate pathway (farnesyl-PP synthase, SQLE, and lanosterol synthase) have been the object of intense investigations as a potential target of therapy. Thus, while HMGCR proved to be the main target of statins, squalene synthase, SQLE, and lanosterol synthase proved to be the targets of several nonstatin inhibitors (lapaquistat, NB-598, and RO48-8071, respectively), none of which yet approved for the clinical use. In addition to these essential enzymes, the transcription factor SREBP2 proved to be a particularly suitable target for therapy due to its regulatory role in the expression of the enzymes involved. Metformin, the most used antidiabetic drug, showed to inhibit SREBP2 activity and indirectly control cholesterol biosynthesis efficiently. Furthermore, of particular interest is the evidence that the combination of this drug with statins considerably reduces the adverse effects caused by the relatively high doses of statins required for prolonged post-surgical adjuvant therapy.

Last but not least, the statins-metform in association could be the winning strategy also in preventing cancer development in healthy women with high mammographic breast density, that is, women whose breasts have a high stroma component often associated with the infiltration of aromatase-overexpressing macrophages, and for this reason at risk of developing breast cancer. Indeed, as demonstrated in a recent study [60], metformin use was associated with a decreased percentage of the dense area and an increase in non-dense areas compared to non-metformin users.

## Abbreviations

17β-HSD1: 17β-hydroxysteroid dehydrogenase type 1
acetyl-CoA: acetyl-coenzyme A
AIs: aromatase inhibitors
BCSS: breast cancer-specific survival
BMI: body mass index
CI: confidence interval
DASIs: dual aromatase-sulfatase inhibitors
ER: estrogen receptor
HMG-CoA: 3-hydroxy-3-methylglutaryl-coenzyme A
HMGCR: 3-hydroxy-3-methylglutaryl-coenzyme A reductase
HR: hazard ratio
IC_50_: half maximal inhibitory concentration
LDL: low-density lipoprotein
RFS: recurrence-free survival
SCAP: sterol regulatory element-binding protein cleavage-activating protein
SQLE: squalene epoxidase
SREBP2: sterol regulatory element-binding protein 2
STS: steroid sulfatase

## Declarations

### Copyright

© The Author(s) 2022.
